# Impact of Dental Anxiety on the Dental Health Status of Nepali Adult Patients

**DOI:** 10.1002/cre2.70034

**Published:** 2024-11-11

**Authors:** Tanuja Singh, Tika R. Ghimire, Manoj Dhungana

**Affiliations:** ^1^ Department of Dentistry Devdaha Medical College and Research Institute Rupandehi Nepal; ^2^ Department of Psychiatry Devdaha Medical College and Research Institute Rupandehi Nepal

**Keywords:** dental anxiety, dental disease, dental fear, dental health status

## Abstract

**Background:**

Patients with high dental anxiety are found to visit dental offices less frequently and have a higher number of severely diseased teeth so they are at a greater need for intensive oral care and rehabilitation.

**Objectives:**

The aim of this study was to assess the prevalence of dental anxiety and its impact on the dental status of Nepali adult patients.

**Material and Methods:**

A semi‐structured questionnaire of the Modified Dental Anxiety Scale (MDAS) in the Nepali version was used for data collection. The data analysis was done using Statistical Package for Social Sciences (SPSS IBM, Chicago, IL, USA, version 24). Variables were calculated as frequency and percentage, while the comparisons of different factors were done using the ANOVA and *t*‐test. A difference with *p *< 0.05 was considered statistically significant.

**Results:**

The study population consisted of 446 subjects, among them 205 were male (45.96%), 241 were female (54.04%), 357 (80.04%) were married, and 222 (49.78%) were employed. The mean age of the patients was 41.24 years (ranging between 18 and 79 years). Only 41 subjects (9.19%) showed high dental anxiety (MDAS ≥ 19). The mean MDAS for the total study population was 10.81. The highest mean MDAS was seen in the age group 30–39 (11.78) and the lowest mean MDAS was seen in the age group 40–49 (9.64). While comparing dental anxiety among genders, dental anxiety was higher in females (mean MDAS = 11.78) compared to males (mean MDAS = 9.67). Patients having high dental anxiety had a significantly higher number of decayed teeth (*p*‐value = 0.001, *t*‐test).

**Conclusion:**

Oral health and dental status both are negatively affected by dental anxiety. It interferes with dental attendance, service delivery, prevention of dental diseases, and early diagnosis. Thus, dental practitioners have a major role to play in the management of dental anxiety.

## 1 Introduction

Psychological problems and mental illness impact the physical well‐being of the person. It acts as a barrier to seeking the required treatment. With an increase in physical disability, the risk of mental illness also increases (Royal College of Psychiatrists 2010; Scott and Happell 2011; Robson and Gray 2007). Anxiety is defined as an aversive emotional state anticipating a feared stimulus in the future, with or without the presence of an immediate physical threat (Armfield 2010a).

Dental anxiety is an intricate, multifactorial phenomenon that consists of unpleasant past dental experiences, negative family attitudes toward dental treatment, blood injury fear, past failure of dental treatment, and fear of injection and pain (Cohen, Snyder, and LaBelle 1982; Locker, Shapiro, and Liddell 1997; Taani 2001). The reluctance of patients with dental anxiety to pursue dental treatment serves as an obstacle to receiving initial preventive care (Crego et al. 2014). In the absence of timely intervention, the severity of the disease worsens resulting in urgent, intensive, and expensive treatment (Berggren and Meynert 1984; Armfield 2013). Patients exhibiting high levels of dental anxiety are found to be making fewer visits to the dentist (Pohjola et al. 2007) and also with more decayed and missing teeth, severe periodontal diseases, and a greater need for intensive oral care and rehabilitation (Klingberg et al. 1995; Pramila and Murthy 2010; Eitner et al. 2006). The extent of dental anxiety may also affect the actual outcome of the treatment. Recognition of anxiety provides crucial information to the dentist to take prior measures to reduce the patient's anxiety for shaping the patient–dentist relationship and successful treatment outcome. Despite advances in technology and pain control in the treatment of dental diseases, dental anxiety remains one of the serious problems that prevent patients from seeking dental treatment on time (Armfield, Stewart, and Spencer 2007).

The percentage of dental anxiety varies from 2% to 30% worldwide depending on the method applied, study population, and cut‐off score used (Smith and Heaton 2003). Besides financial constraints, dental anxiety is also one of the major factors in avoiding dental treatment and appointments. In the context of Nepal, very little research has been done regarding dental anxiety. There are studies carried out to evaluate dental anxiety in parents accompanying children for dental treatment, the prevalence of dental fear among school children, in patients undergoing oral surgical procedures, and also among groups of dental students but to our knowledge from the indexed literature no study has been done to analyze dental anxiety and its impact on dental status in Nepali adult patients. This study aims to assess the prevalence of dental anxiety and its impact on the dental status of adult patients.

## 2 Materials and Methods

### 2.1 Study Design and Sampling

An observational, cross‐sectional study was conducted among patients attending a dental surgery clinic. The study sample consisted of 446 patients (205 men and 241 women) who visited the dentist between April 2022 and October 2022. The purpose of the study was explained to the patients and written consent was obtained. Inclusion criteria were patients aged 18 years or older who consented to the study and agreed to complete the questionnaire. Exclusion criteria were age below 18 years or patients unable to understand the Nepali questionnaire. Dental status was recorded as DMFT score by examining the patient's oral cavity. Ethical clearance for the research was secured from the ethical approval committee at Devdaha Medical College and Research Centre. (Ref no: 573/078/079).

### 2.2 Questionnaire

A semi‐structured questionnaire, based on the Modified Dental Anxiety Scale (MDAS) and translated into Nepali with permission from the principal author, was utilized as the data collection tool (Giri et al. 2017). The Nepali version of the MDAS is a 5‐question survey that evaluates the dental anxiety levels of people and how they feel about different dental procedures. All questions have a consistent answering pattern for each question in the Likert scale ranging from “not anxious” to “extremely anxious.” Hence, it is simple to understand and consumes less time to complete. Patients were asked to fill out the questionnaire in the waiting area. Each response is scored from 1 to 5. It is added together with a minimum score of 5 and a maximum score of 25 on the Likert scale with a cut‐off score of 19 and above indicative of significant dental anxiety (Humphris et al. 2013). Moreover, it is crucial to note that completing the questionnaire does not increase the patient's anxiety levels (Humphris and Hull 2007).

### 2.3 Clinical Examination

Once the questionnaire was complete, the principal investigator examined the oral cavity of the patient using a plane mouth mirror and explorer under the adequate amount of light for decayed (D), missing (M), and filled (F) teeth (DMFT) index according to the WHO guidelines (World Health Organization 2013). Dental radiographs and third molar were not included in the study. Decayed teeth (D) were characterized by the presence of primary and secondary caries. Missing teeth (M) were identified by the absence of teeth, regardless of the cause. Filled teeth (F) encompassed teeth filled with various materials, including crowns. The examiner documented the individual counts of D, M, and F for each participant, with their sum yielding the DMFT score. This score serves as an indicator of dental disease and prior dental interventions up to the examination date.

### 2.4 Statistical Analysis

The data were analyzed using Statistical Package for Social Sciences (SPSS IBM, Chicago, IL, USA, version 24). Variables were calculated as frequency and percentage, while the comparisons of different factors were done using the ANOVA test and *t*‐test. Differences with *p* < 0.05 were considered statistically significant.

## 3 Results

The study population consisted of 446 subjects, among them 205 (45.96%) were male, 241 (54.04%) were female, 357 (80.04%) were married, and 222 (49.78%) were employed. The mean age of the study population was 41.24 years (range 18–79 years). Only 41 (9.19%) subjects showed high dental anxiety (MDAS ≥ 19).

**Figure 1:**
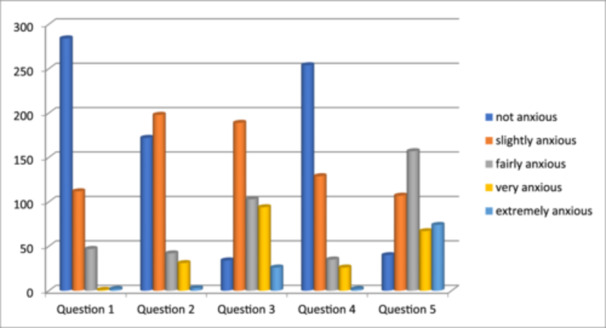
Bar diagram representation of patient response to anxiety level in different dental situation.

Table 1 shows the modified dental anxiety scores among different age groups. The age group of the study population ranged from 18 years and above. The mean MDAS for the total study population was 10.81. The highest mean MDAS was seen in the age group 30–39 (11.78) and the lowest mean MDAS was seen in the age group 40–49 (9.64). The mean MDAS score was found to be higher in the younger age group than in the older age group, which was statistically significant (*p *< 0.001, ANOVA test). This suggests that the younger age group population has a higher dental anxiety than the older age group population. It was found that women experience significantly higher dental anxiety than men (mean MDAS: 11.78 vs. 9.67), with more women (6.95%) falling into the “highly anxious” category compared to men (2.24%) as depicted in Table 2. Both genders shared similar fears, with injections and drilling being the most anxiety‐provoking (Table 3). Interestingly, people with high anxiety had significantly more cavities (*p *< 0.001), but no significant differences in missing teeth, filled teeth, or overall dental health scores (Table 4). This suggests a possible link between anxiety and cavities, but not necessarily other dental problems. The reliability of questionnaires in all samples was calculated by Cronbach's alpha which was 0.85 showing good internal uniformity. The responses of the participants to all the questionnaires are illustrated in Figure 1, which shows that maximum participants are not anxious for Questions 1 and 4 that is if they have an appointment tomorrow or they were about to have teeth scaled and polished; however, in the case of receiving local anesthesia in gum (Question 5), most of the participants were fairly anxious.

**Table 1:** Means, standard deviation (SD), and percentages of dental anxiety by age group including patients with high dental anxiety (MDAS ≥ 19).

Age groups (Years)	*N*	%	Mean	SD	High Dental anxiety MDAS ≥ 19%	
					*N*	Within age group
18–29	135	30.27	11.61	4.19	23	10.04
30–39	87	19.51	11.78	4.27	13	14.94
40–49	73	16.37	9.64	3.09	3	4.11
50–59	64	14.35	10.14	2.95	0	0
60–69	74	16.59	10.05	4.48	1	1.35
≥ 70	13	2.91	10.31	3.86	1	7.69
Total	446	100	10.81	3.84	41	9.87
*p*‐value (ANOVA)				< 0.001		

**Table 2:** Means, standard deviation (SD), and percentages of dental anxiety by gender, including patients with high dental anxiety (MDAS ≥ 19).

Gender	*N*	%	Mean	SD	High Dental anxiety MDAS ≥ 19%	
					*N*	Within gender
Male	205	45.96	9.67	3.44	10	2.24
Female	241	54.04	11.78	3.89	31	6.95
Total	446	100	10.81	3.84	41	9.87
*p*‐value (*t*‐test)				< 0.001		

**Table 3:** Single item MDAS mean according to gender.

Questionnaire item (MDAS)	Male	Female	*p*‐value
If you went to your dentist for Treatment Tomorrow, how would you feel?	1.33	1.62	0.000
If you were sitting in the Waiting Room, how would you feel?	1.63	2.07	0.000
If you were about to have a Tooth Drilled, how would you feel?	2.50	2.97	0.000
If you were about to have your Teeth Scaled and Polished, how would you feel?	1.43	1.81	0.000
If you were about to have a Local Anesthetic Injection in your gum, how would you feel?	2.79	3.31	0.000
Total mean score according to gender.	9.67	11.78	0.000

**Table 4:** Mean decayed, missing, and filled permanent teeth with low and high dental anxiety.

Variable	Low dental anxiety MDAS < 19	High dental anxiety MDAS ≥ 19	*p*‐value
Decayed(D)	2.36	4.17	0.000
Missing(M)	1.51	0.95	0.196
Filled(F)	1.86	1.27	0.068
DMFT	5.74	6.39	0.327

## 4 Discussion

The terms dental anxiety, phobia, and fear are used interchangeably, although they differ depending upon the situation within which they occur. Dental anxiety typically arises in response to an anticipated, yet unidentified threat, whereas dental fear or phobia emerges in reaction to a recognized danger, often prompting a “fight‐or‐flight” response upon encountering a perceived threatening stimulus (Armfield 2010b). Despite advancements in technology and pain‐free dental procedures, dental anxiety still has a deterrent effect on patients seeking dental care.

Prevalence of high dental anxiety (MDAS ≥ 19) was found in 9.8% of the population whereas it was found only in 2% of the population who reported to the Department of Orthodontics, B.P. Koirala Institute of Health Sciences (BPKIHS), Dharan, Nepal (Giri et al. 2017). However, various studies have shown that it ranges between 5% and 20%. It varies among various social and cultural backgrounds such as India (5.9%) (Appukuttan et al. 2017), China (8.7%) (Yuan et al. 2008), Saudi Arabia (8.5%) (Akeel and Abduljabbar 2000), United Kingdom (11.6%) (Humphris, Dyer, and Robinson 2009), Netherland (17.9%) (Stouthard and Hoogstraten 1990), Australia (9.5%) (Armfield, Slade, and Spencer 2009), and USA (12.2%) (Coolidge et al. 2010).

In this study, a statistically significant relationship between age and high dental anxiety was observed. The findings suggest that dental anxiety tends to decrease with advancing age, which is similar to that of a study conducted from 1969 to 1996 by Hägglin et al. (1999) which states that dental fear, like numerous other general and specific phobias, declines with age. In our study, the lowest anxiety was seen in the age group 40–49 years, and this may be due to the reason they have ample time to experience good dental treatment and neutralize the previous bad experiences. In the more older population, high dental anxiety is seen more than in the middle‐aged population and less than in the younger age. This may be because the older age population has gone through more dental treatment experiences than the younger age but aging is predestined to degradation in health and an increase in the need for physical, financial, and emotional support which affect the overall well‐being of individuals (Awang et al. 2018). There will be an increase in mental disorders with a degradation in health (Ma et al. 2021).

The results of this study showed a statistically significant higher level of dental anxiety in women than in men. This finding aligns with numerous other studies examining dental anxiety among genders, consistently indicating a higher prevalence of anxiety among females (Humphris, Dyer, and Robinson 2009; Khraisat and Al‐Olaimat 2013). This may be due to the difference in anxiety levels in males and females, moreover, females are more willing to accept their anxiety and are reluctant for dental treatment than males.

In this study, we found that individuals with high levels of dental anxiety tended to have a greater number of decayed teeth in comparison to those with low levels of dental anxiety. This observation is similar to the study by Eitner et al. (2006) and Khraisat and Al‐Olaimat (2013). This may be due to the avoidance of regular dental visits by a patient with high dental anxiety, which results in increased caries morbidity. Patients with high dental anxiety do not make regular dental appointments and thus the dental professional cannot diagnose and treat the carious lesion during the initial stage to avert the progression of tooth decay. However, the relationship between missing teeth (MT) and filled teeth (FT), as well as overall DMFT among the highly anxious patients and the low anxious patients was not statistically significant. Patients with low dental anxiety had more missing and filled teeth compared to those with high dental anxiety in contrast to the study done by Zinke, Hannig, and Berth (2018). This may be due to the reason that patients with low dental anxiety visit the dentist more frequently but in a country like Nepal with low socioeconomic income, patients prefer extraction over other expensive restorative or rehabilitative treatment procedures like root canal treatment due to financial reasons.

For this, an extensive study is required as to why people with low dental anxiety have more missing teeth as in this study socioeconomic status was not taken into consideration. The clinical data in this study are acquired solely through clinical observations. For the patients with no previous dental records, the clinician had to exclusively depend on the patient's history for the reason of tooth loss as tooth loss due to orthodontic treatment and accident should not influence the index of oral health (Lesaffre, Mwalili, and Declerck 2004). Observer bias in detecting the caries was eliminated since only the principal investigator solely did clinical examinations.

## 5 Conclusion

Dental anxiety is one of the most common impediments to dental treatment and many studies have been done on this matter, but this study assesses dental anxiety through the Nepali version of MDAS and DMFT index in patients. This study also assesses the connection between dental anxiety in relation to age and gender. In general, dental anxiety has a negative effect on the execution of dental services. Patients with high dental anxiety tend to avoid regular dental visits until the oral health problem intolerably agonizes them, which will increase the morbidity and cost of treatment. Thus, a dentist needs to recognize the problem in an early stage, be patient and comforting, and have good communication and behavior management skills. Patients also should recognize their fear of dental visits and procedures and let the dentist know about the same. Avoiding unpleasant procedures on the first visit patients can have pleasant dental experiences.

## Author Contributions

*Conceptualization*: Tanuja Singh. *Methodology*: Tanuja Singh and Tika R. Ghimire. *Investigation*: Tanuja Singh and Tika R. Ghimire. *Formal analysis*: Tanuja Singh and Tika R. Ghimire. *Writing–original draft*: Tanuja Singh. *Writing–review and editing*: Tika R. Ghimire. *Resources*: Tanuja Singh and Tika R. Ghimire. *Funding acquisitions*: Tanuja Singh, Tika R. Ghimire, and Manoj Dhungana. *Project administration*: Tika R. Ghimire and Manoj Dhungana.

## Conflicts of Interest

The authors declare no conflicts of interest.

## Data Availability

See https://doi.org/10.6084/m9.figshare.22354831.
